# Complementary feeding methods in the first year of life: a study protocol for a randomized clinical trial

**DOI:** 10.1186/s13063-021-05647-1

**Published:** 2021-10-09

**Authors:** Leandro Meirelles Nunes, Jordana Führ, Christy Hannah Sanini Belin, Paula Ruffoni Moreira, Renata Oliveira Neves, Mariana Lopes de Brito, Lorenzo Andreazza Morando, Adriela Azevedo Souza Mariath, Erissandra Gomes, Juliana Rombaldi Bernardi

**Affiliations:** 1 Graduate Program in Child and Adolescent Health, Universidade Federal do Rio Grande do Sul (UFRGS) Medical School, Porto Alegre, Brazil; 2 Neonatology Section, Hospital de Clínicas de Porto Alegre (HCPA), Porto Alegre, Brazil; 3 Graduate Program in Food, Nutrition and Health, Universidade Federal do Rio Grande do Sul (UFRGS) Nutrition Department, Medical School, Porto Alegre, Brazil; 4 Department of Surgery and Orthopedics, Universidade Federal do Rio Grande do Sul (UFRGS) Dentistry School, Porto Alegre, Brazil; 5 Nutrition Department, Universidade Federal do Rio Grande do Sul (UFRGS), Hospital de Clínicas de Porto Alegre (HCPA), Porto Alegre, Brazil

**Keywords:** Complementary feeding, Feeding behavior, Feeding preferences, Infant overweight, Iron deficiency anemia, Choking, constipation, Clinical trial

## Abstract

**Background:**

The traditional spoon-feeding approach to introduction of solid foods during the complementary feeding period is supported by consensus in the scientific literature. However, a method called Baby-Led Introduction to SolidS (BLISS) has been proposed as an alternative, allowing infants to self-feed with no adult interference. To date, there have been no trials in the Brazilian population to evaluate the effectiveness of BLISS in comparison to the traditional approach.

**Methods/design:**

To evaluate and compare three different complementary feeding methods. Data on 144 mother-child pairs will be randomized into intervention groups by methods: (A) strict Parent-Led Weaning; (B) strict Baby-Led Introduction to SolidS; and (C) a mixed method. Prospective participants from Porto Alegre, Brazil, and nearby cities will be recruited through the Internet. The interventions will be performed by nutritionists and speech therapists, at 5.5 months of age of the child, at a private nutrition office equipped with a test kitchen where meals will be prepared according to the randomized method. The pairs will be followed up at 7, 9, and 12 months of age. Data will be collected through questionnaires designed especially for this study, which will include a 24h child food recall, questionnaires on the child’s and parents’ eating behavior, oral habits, eating difficulties, and choking prevalence. At 12 months of age, children will undergo blood collection to measure hemoglobin, ferritin, and C-reactive protein, saliva collection for analysis of genetic polymorphisms, and oral examination. Anthropometric parameters (child and maternal) will be measured at the baseline intervention, at a 9 month home visit, and at the end-of-study visit at the hospital. The primary outcome will be child growth and nutritional status *z*-scores at 12 months; secondary outcomes will include iron status, feeding behavior, acceptability of the methods, dietary variety, choking, eating behaviors, food preferences, acceptance of bitter and sweet flavors, suction, oral habits, oral hygiene behavior, dental caries, gingival health status, and functional constipation.

**Discussion:**

The trial intends to ascertain whether there are potential advantages to the BLISS complementary feeding method in this specific population, generating data to support families and healthcare providers.

**Trial registration:**

Brazilian Clinical Trials Registry (ReBEC): RBR- 229scm number U1111-1226-9516. Registered on September 24, 2019.

## Background

Complementary feeding (CF) is the process of introducing solid and liquid foods other than breast milk or infant formula to an infant’s diet [1]. The CF period represents the transition from milk feeding to solid foods [2]. Over the past half-decade, researchers have started to conduct studies about specific approaches to CF [3–10]. In the last decade, an alternative approach named Baby-Led Weaning (BLW) which consists of allowing the infant to make their own food choices, deciding the quantity and sensory characteristics of food according to their own energy needs, has been widely disseminated through digital media [11]. This method suggests offering food in pieces, strips, or sticks and claims that CF should not be influenced by the imposition of caregivers, who generally assume what and how much food the child needs and wants [12]. Currently, the Brazilian guideline recommends traditional spoon-feeding based on responsive feeding and suggests that soft foods can be offered to the child to handle if they are large pieces, and that meals have their consistency gradually progressed until, at twelve months of age, they reach a food consistency used by the child family [13].

Considering several questions raised by parents and healthcare professionals regarding the BLW method, such as possible risks of choking and inadequate supply of iron and calories, a group of New Zealand professionals created a modified version of this approach, which they named Baby-Led Introduction to SolidS (BLISS). The BLISS recommendations are the same as those of the BLW method, with some additional advice, including offer food shaped in large pieces, which the infant can hold by himself; guarantee one source of iron-rich food at each meal; offer a high-calorie food at each meal; offer prepared foods in a texture that reduces the risk of choking, and avoid foods listed as high risk of aspiration; always try the food before offering it to the infant, to check it does not become solid in the oral cavity; avoid round or coin-shaped foods; and ensure that the infant is seated, erect, and under supervision of an adult. A study compared the BLW and BLISS approaches, where BLISS participants received two intervention visits, resources, and on-call support, while BLW participants received no intervention. Findings showed that the BLISS intervention allowed to increase the offerings and variety of iron-containing foods and to reduce the offering of high-choking-risk foods [14].

Although the Brazilian guideline for CF has been reviewed and republished recently, the conventional method, Parent-Led Weaning (PLW), is still advised, and there are no recommendations for a baby-guided approach in Brazil [13]. Likewise, the current World Health Organization (WHO) guidance proposes pureed foods in the early stages of CF, with consistency progressing gradually along the first year of life [15]. Within this context, the present study intends to contribute to the following outcomes:

### Growth faltering

A nutritionally adequate diet is essential throughout childhood and especially crucial in the first 3 years of life, which are a critical period for growth faltering [13, 15]. Previously known as failure to thrive, growth faltering is defined as a current weight or rate of weight gain lower when compared to those of average children of the same age and sex [16]. It is related to many organic and nonorganic causes, including oral and motor function, which has raised concerns among healthcare professionals as to how efficiently an infant would be able to self-feed in terms of quantity as well as how motivated an infant would be to lead the feeding process [17].

It is still unclear whether a Baby-Led method would interfere with the growth process. The evidence available to date has not found any children with faltering growth, nor any significant differences in growth rate between the CF groups at 12 months, although recommendations about offering a high-calorie food with each meal were given [18]. Another study was designed to analyze this outcome [19], however, did not use the BLISS recommendations for energy intake, and assessed different ages. A case-controlled study examined children aged 20 to 78 months and found that spoon-fed ones appeared to be more obese (*z*-score > + 3), while the incidence of underweight (*z*-score < − 3) was higher in the BLW group [9]. A year after this study, a longitudinal investigation of self-reported weight in a sample aged 18 to 24 months found that children weaned with the standard method were more likely to be overweight (19.12% versus 8.1% in the BLW group) [20].

### Iron deficiency

Infants are vulnerable to anemia, especially iron-deficiency anemia (IDA), which represents a major public health problem with definite impacts on population health [21]. The literature suggests that early eating practices play an important role in the prevalence of iron deficiency [22]. Anemia is defined as the condition in which the blood hemoglobin concentration is two standard deviations below the reference for age, sex, and height [23]; 50% of cases are due to iron deficiency. It is estimated that about 25% of Brazilian children under age 3 years have IDA, and the promotion of breastfeeding, healthy eating habits, and prophylactic iron supplementation have long been employed as strategies to prevent this condition [24].

BLISS method does not appear to increase the risk of iron deficiency in comparison to PLW when parents are advised to offer high-iron foods in each meal [25]. Given the evidence of IDA in this age group and the scarcity of published studies on infant-guided feeding introduction methods (none in the Brazilian population), systematic investigation and evaluation of anemia in early childhood are particularly important.

### Feeding behavior

The feeding styles of caregivers and choice of CF method may influence the complex process of eating behavior [26, 27]. Caregivers’ encouragement of healthy eating and the responsibility and supervision of feeding was associated with reasonable children’s eating behaviors, especially low children’s emotional eating, infrequent satiety responsiveness, and food fussiness behaviors [27]. The authoritative style of parenting has a significant negative direct effect on children’s including emotional overeating and food fussiness [28]. Additionally, high parent-centered feeding along with high child-centered feeding styles was associated with improved diet quality and the lowest percentage of overweight and obese children in a study of Latino families [29].

A study of 565 parents with toddlers aged 12 to 36 months in the United Kingdom found that parents who were following a strict, predominantly BLW style used less instrumental feeding practices, exerted significantly less control over their toddler’s eating, used significantly less encouragement to increase food consumption, shared their mealtimes, and ate the same meals with their children [30]. The food preferences developed at this age have a long-lasting impact. Combined with the stress surrounding infant feeding decisions, feeding advice for parents must be aligned with appropriate expectations regarding effects on eating behavior.

### Dietary quality

A Baby-Led approach to complementary feeding has been suggested to increase dietary variety and exposure to more textured foods at a young age. According to Morison et al. (2018), at 7 months of age, BLISS participants had greater total food variety with no difference in fruit and vegetable variety. However, at 24 months of age, BLISS children had greater variety in fruits and vegetables compared to traditionally spoon-fed children [31].

BLW researchers suggest that a baby-guided approach leads to healthier food choices, since there is greater fruit and vegetable exposure and variety, as well as greater participation in family meals [4, 32]. However, few studies have investigated whether baby-guided approaches influence food variety [3, 8, 33].

### Feeding difficulties

The prevalence of feeding difficulties in childhood ranges from 20 to 35% [34] and represents a high-impact clinical problem for children and families alike [35]. The consequences of these difficulties include growth faltering, malnutrition, lethargy, developmental delay, aspiration, invasive medical procedures (such as feeding tube placement), and hospitalization [36].

Children with feeding difficulties, such as food neophobia or picky eating, consume fewer fruits and vegetables, have a lower-variety diet, and weigh in at lower percentiles [37]. In addition to their potential impact on physical well-being and development, eating problems are associated with parental stress, emotional well-being, and parent-child interactions [38]. To the best of our knowledge, the evidence published to date has not yet analyzed the influence of different CF approaches on the prevalence of feeding difficulties.

### Food preferences

The strongest predictors of food preferences in childhood are whether the child likes how food tastes; how long they were breastfed, and, while breastfeeding, what their mothers were eating; and whether they were offered and ate these foods at an early age [39]. Therefore, food choices are strongly associated with sensory properties, leading to acceptance or aversion to certain foods [40]. The sensation of flavor is formed by a complex combination of taste, smell, and somatosensory information [41].

Variations in taste perception among individuals can also shape flavor preferences, influencing dietary intake and long-term eating habits [42]. Studies have demonstrated that genetic variations related to bitter taste, for instance, may be related to acceptance of feeding introduction [43], to food selectivity [44], and higher daily energy density from snacks in children aged 3 to 5 years [45]. Regarding sweet taste, research has related the TAS1R2 and TAS1R3 receptors to changes in preference for sweet taste and eating behavior, higher risk of cavities, and greater consumption of carbohydrate-rich foods [45, 46]. However, no studies have explored the impact of different forms of feeding introduction and environmental modulation on acceptance of specific flavors in childhood while analyzing associated genetic polymorphisms as well.

### Oral habits

The prevalence of dental caries in individuals up to 3 years of age in different Brazilian cities has been reported to range from 11 to 29% [47, 48]. Sucrose is known to be an essential risk factor for the development of this disease [49]. The age of onset of sucrose exposure appears essential; dietary factors observed before age 12 months are associated with severe early childhood caries (S-ECC) at preschool age [50, 51]. Duration and type of breastfeeding also appear to influence the experience of dental caries [51, 52].

Otherwise, results of a study in which researchers used electromyography suggest that the muscle activity of infants who are breastfed exclusively renders them less likely to develop dysfunctional muscular patterns that might predispose bottle-fed infants to develop a malocclusion [53].

A study that compared baby-led weaning with the traditional method demonstrated that the former was associated with lower odds of exposure to sucrose and processed foods [54], which could contribute to better oral outcomes. Therefore, our intention in evaluating oral habits is to investigate whether feeding methods could lead to different oral outcomes in our population. We are not aware of any published literature on the effects of baby-guided CF on dental caries or gingival inflammatory status.

### Choking

Parents, caregivers, and health professionals often express concern regarding the risk of choking associated with CF methods, especially the BLW. First, it is prudent to differentiate choking/asphyxiation from the gag reflex, which is a normal physiological condition. In a review that addressed the theme, there was no difference in the proportions of choking/asphyxiation between those who adhered to the BLW strategy and those exposed to traditional eating behavior, despite the fear in the discourse of parents and health professionals [55]. Clinical studies also found no difference and reported a small number of severe choking/asphyxiation events in both groups [56, 57]. Regardless of the CF method, it is important to emphasize that parents/caregivers should offer safe food in appropriate environments, as well as to know how to correctly manage babies in case of choking [56].

### Functional constipation

Functional constipation (FC) is a frequent complaint in pediatric clinics, with a worldwide prevalence in children ranging from 0.5 to 32.3% [58]. In addition to impacting the quality of life of these children and their families, the process of diagnosis and treatment of FC is financially demanding on health systems [59]. Factors that can contribute to FC include pain, fever, dehydration, food and fluid intake, psychological issues, toilet training, medications, and family history of constipation [60]. The lack of data in the literature on the impact of different CF methods on the amount of fiber ingested and the consequences of this intake for intestinal health makes the study of this outcome important to guide future recommendations.

## Methods/design

### Study design

**Fig. 1:**
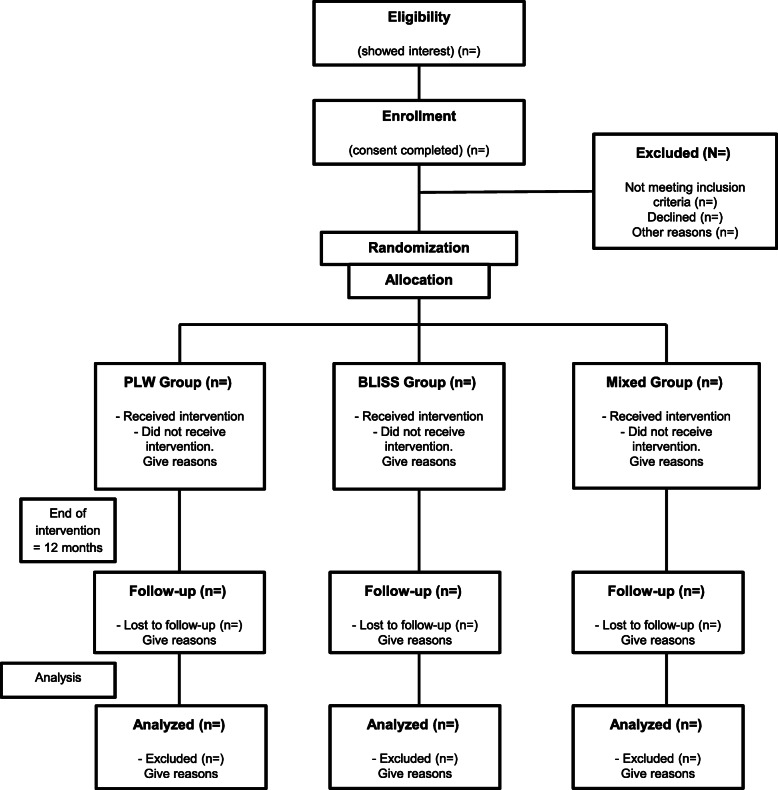
Study design flow chart. PLW, Parent-Led Weaning; BLISS, Baby-Led Introduction to SolidS. Adapted from Daniels et al. (2015) [18]

The study will consist of a three-arm controlled trial (Fig. 1), which will intend to randomize mothers and their respective infants into three different groups of CF: (A) strict Parent-Led Weaning (PLW), which is the control arm; (B) strict Baby-Led Introduction to SolidS (BLISS); and (C) mixed method: a combination of PLW and BLISS created especially for this study. Three interventions will be performed throughout the study, and measurement of the main outcomes is expected at 12 months of age. The framework expected to the outcomes is that BLISS and mixed methods will be superior to the PLW method.

This study has been approved by Hospital de Clínicas de Porto Alegre (HCPA) Ethics Committee (under number 2019-0230, CAAE: 1537018500005327) and is currently registered in the Brazilian Clinical Trials Registry (ReBEC) with accession number RBR- 229SCM. The protocol was written according to SPIRIT guideline, and its checklist was carried out (Additional File).

Written informed consent will be obtained from all responsible of the participants before randomization. This document specifies all steps carried out in the research, as well as the consent to publish the data and the confidentiality, making it clear that the results will be presented together, without identifying or naming the participants. The research team will clarify all participants’ questions about the research procedures before signing the consent form. The research will bring direct benefits of nutritional support to participant children, as well as clarify any doubts expressed by mothers at the start of the study and throughout the study period, until age 12 months. A survey will provide feedback on nutrition assessment results and laboratory tests, by e-mail or letter; in case of relevant changes, a copy will be forwarded to the referral health unit. If any nutritional deficit or excess is detected, the child will be referred to an appropriate health care provider.

### Participants and recruitment

Participants will consist of parents and their lactating children. Recruitment will take place by online invitation, through social networking pages, targeted mothers’ groups, and the HCPA bulletin board. The invitation letter will contain a phone number and an email address where parents can leave a message if they wish to participate. Once checked against the inclusion criteria, those found eligible will receive a standardized message explaining the study details, risks, and benefits, and any further questions will be answered by the researchers by phone or e-mail. After confirmation of interest, the consent form will be sent by email. Once the signed consent form is received back, the participant will be randomized to one of the groups.

### Inclusion criteria

Mothers living in Porto Alegre, Rio Grande do Sul, Brazil (population 1,483,771) [61] or nearby cities, with healthy singleton infants born at term, with birth weight ≥ 3500 g, between 0 and 4 months of life and who have not yet started the CF process will be eligible to participate.

### Exclusion criteria

Children who develop neurological deficits or congenital abnormalities, or any food restriction (as indicated by their pediatrician) during the follow-up period will be excluded from the study.

### Sample size

The sample size was calculated using WinPepi® software version 11.65, based on previously published studies on the subject. Considering a unit standard deviation of 1, statistical power of 80%, and a significance level of 5% to detect a difference in BMI of 0.8 kg/m^2^ [18, 24], the sample calculation for a half standard deviation difference will consist of 48 mother-infant pairs for each of the three intervention groups, totaling 144 pairs of mothers and infants.

### Randomization

After signing the consent form, participants will be sequentially numbered and have their names entered into a randomization list of three blocks and equal numbers, previously computer-generated (http://www.randomization.com) by blinded research staff.

Only at the time of the first intervention will mothers be aware of their group allocation. Assessment data will be collected by a different researcher from the one responsible for the intervention, blinded to the allocation group, in a different room.

### Study groups

All participants will receive one of three interventions during the study. First, when infants are 5.5 months old, a dietary workshop at a private nutrition office equipped with the test kitchen, in which nutritionists will teach parents how to begin CF as appropriate for the group to which they were randomized. Participants and nutritionists will cook example meals together in real-time at the test kitchen (Table 1).

**Table 1:**
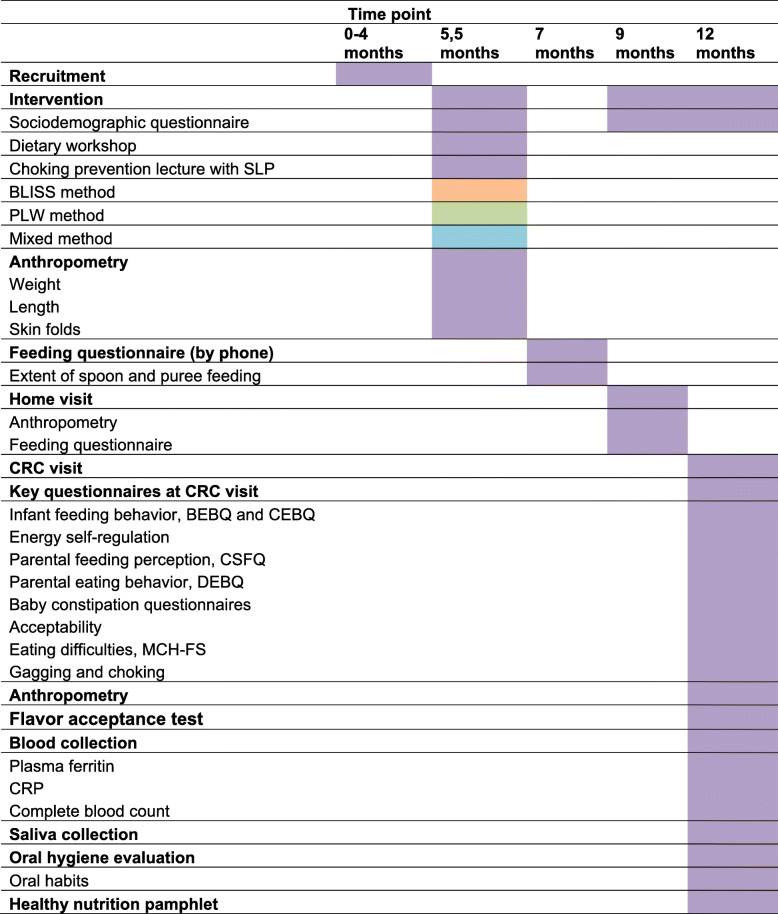
Interventions and outcome measures at specified time points

*BLISS*, Baby-led Introduction to SolidS; *PLW*, Parent-Led Weaning, *CRC*, Clinical Research Center; *CRP*, C-reactive protein; *BEBQ*, Baby Eating Behavior Questionnaire; *CEBQ*, Child Eating Behavior Questionnaire; *CFSQ*, Caregiver Feeding Style Questionnaire; *DEBQ*, Dutch Eating Behavior Questionnaire; *MCH-FS*, Montreal Children’s Hospital Eating Scale; *SLP*, Speech-Language Pathologists

At the same visit, speech therapists will advise on choking prevention, showing videos with examples of choking and gagging. All advice will be standardized; each group will have its own set of printed material of topics to be addressed by the professional, and each participant will receive a copy to take home. The major differences between the intervention groups are the consistency of foods and the level of adult participation during each meal. The basic information that every group will receive during the first intervention is:

- Encouragement to breastfeed exclusively for 6 months and continue breastfeeding to at least 24 months or longer.
- Not to start CF until their infant is 6 months of age.
- Make sure the infant is always sitting upright and under adult supervision when they are eating.
- Specific food techniques to improve food bioavailability of iron and energy.
- Avoid offering honey, sugar, caffeine, juice, processed foods, and low-energy foods, such as soups, in the first 2 years of life.
- At age 6 months, offer a fruit at two different times plus one main meal (lunch) and, by 7 months, introduce a second main meal (dinner).
- Not to sift and/or liquefy any type of food.
- Not to offer hard foods such popcorn or nuts, nor any foods that might become sticky in the mouth, such as peanut butter.

After the first intervention, every mother will receive a dietitian’s personal phone number to call if they have questions related to the study or the CF process.

Second, a home visit at 9 months of age will serve to measure anthropometric parameters of the mother and child. At this time, questionnaires will also be administered and any extra individualized advice or support for the CF process will be provided, always appropriate for the approach to which each mother-child pair was allocated. The visits will be led by two blinded researchers; to avoid unblinding, one will obtain anthropometric measurements before the other applies the questionnaire, as some answers given might reveal the allocation group. The visit will end with a thank-you card, to motivate family engagement with the study.

Finally, the last intervention will be conducted at the HCPA Clinical Research Center (CRC). Blood samples will be drawn for measurement of plasma ferritin, C-reactive protein (CRP), and a complete blood count, as well as saliva for genetic tests. Anthropometric measurements will be repeated, an intraoral examination will be performed, and the end-of-study questionnaires will be administered, as well as a flavor preference test. Mothers will then receive a booklet with advice on healthy nutrition and oral habits, such as nutritional assessment, oral evaluation, and oral hygiene guidance; blood test results; and nutritional guidelines for the child’s further years to come, encouraging them to continue to pay attention to healthy eating.

### Parent-Led Weaning group

Mothers allocated to the PLW group, besides the standard information described above, will be encouraged to spoon-feed pureed foods to their infants, following the traditional feeding method. Consistency should progress gradually until family meal texture is reached by 12 months of age. Mothers will be instructed not to mix or incorporate more than one food at a time, to ensure the child is capable of learning the difference between flavors.

### BLISS group

In addition to the standard information, participants from the BLISS group will be taught to prepare meals shaped into sticks, allowing the child to self-feed without adult interference. The ideal food consistency, size, and shape will be demonstrated during the workshop to guarantee the method is properly performed. Recipes for adaptation of foods such as beans will be given and explored. Although we will not expect the adult to feed the child, we intend to emphasize the difference between not interfering with the feeding process and supervising the child, with an emphasis on the advice to never leave the child alone during a meal.

### Mixed group

This group will be instructed to combine the two methods explored above according to the child’s wishes. In short, the mother would prepare meals in both consistencies and allow the child to both be spoon-fed and grab sticks of food concomitantly as they desire, without following a regular pattern. If the child prefers to eat certain types of foods as sticks and others mashed, that would be allowed, i.e., self-feed fruit sticks and then be spoon-fed dinner. All standard information would also be given to this group.

### Adherence

To ensure the families follow these instructions, a blinded researcher will conduct a phone call when the child is 7 months of age and ask some key questions (about food consistency when the child started to eat, and at 7 months; if the child takes food to mouth alone; and if the child receives food in spoon). A different researcher will analyze the answers and compare them with further data collected at the 9-month visit, in addition to a 24-h dietary record. These records will be collected during the home visit, by trained researchers. However, as some mothers might not be able to quantify food intake exactly, all participants will also be asked when they introduced a variety of different foods at the 9-month and 12-month visits. Questionnaires to assess adherence to self-feeding, breastfeeding practices, and introduction of complementary foods at 6 months will also be administered at the 9-month and 12-month visits. The rates of adherence will be analyzed. All participants will be included to the final analyses, regardless of having followed the method, since the analyzes will be made by intention-to-treat.

### Outcome measures

*Z*-scores of anthropometric measurements (weight for age, weight for length, length for age, and BMI for age *z*-scores) will be our primary outcome measure to investigate child growth and nutritional status. As secondary outcomes, we will analyze choking prevalence, dietary variety, food and flavor preference, child and parent feeding behavior, iron deficiency, oral hygiene behavior, dental caries, dental development, gingival health status, the prevalence of functional constipation, maternal perception of the CF methods, and prevalence of child eating disorders.

### Anthropometric measures

The first questionnaire, administered at 6 months, will inquire about birth weight, birth length, and head circumference. Mothers are expected to obtain these data from the child’s hospital records or vaccination card.

At 6, 9, and 12 months of age, blinded researchers trained by the International Society for the Advancement of Kinanthropometry (ISAK) [62] will obtain anthropometric measurements, double-checking and calculating a mean value for every assessment. Inter-observer technical error of measurement (TEM) [63] and the coefficient of reliability will be calculated, and anthropometry standardization sessions will be conducted to monitor the measurement techniques and prevent error or bias. Teams will be retrained throughout the study as necessary.

All infant participants will be weighed completely undressed and diaperless, in the laps of their mothers, who will be expected to wear light clothes only (no shoes, cellphone, wallets, or other accessories). The mothers will then be weighed alone, and the latter result subtracted from the former to obtain the child’s weight. We intend to use calibrated digital scales (Plenna®) with 0.1 kg resolution.

Recumbent length will be measured to the nearest 0.1 cm using a portable length board. The same calibrated equipment will be used at every phase of the study to ensure accuracy. All measurements will be taken in duplicate and the average calculated. BMI (kg/m^2^) and BMI-for-age (*z*-score) at 6, 9, and 12 months of age, as well as weight-for-age, weight for length, and length for age *z*-scores, will be calculated using the WHO child growth standards [16].

Skinfolds (triceps and subscapular) will be measured with a calibrated adipometer (Lange®) in mothers and infants, as well as arm and waist circumference in mothers and head and arm circumference in children.

### Questionnaires

Upon study enrollment, mothers will complete a sociodemographic questionnaire for calculation of the Brazilian Index Score (ABIPEME criteria) [64].

At 9 and 12 months, we will investigate feeding practices and the presence of choking. Eating behaviors and intestinal habits will be inquired specifically at the 12-month visit, as will the acceptability of the allocated CF approach to parents, focusing on “mess”, convenience, and cost.

Food intake will be assessed by a 24-h dietary recall. Data on oral hygiene habits will be collected through a questionnaire created for this study. Infant feeding behavior will be tested by the Baby Eating Behavior Questionnaire (BEBQ) [65] and Children Eating Behavior Questionnaire (CEBQ) [66]. Parental feeding perception will be assessed by the Caregiver Feeding Style Questionnaire (CFSQ) [67], while parental eating behavior will be assessed by the Dutch Eating Behavior Questionnaire (DEBQ) [68]. Finally, eating difficulties will be assessed with the Montreal Children’s Hospital Eating Scale (MCH-FS) [69].

### Choking

At the 9-month and 12-month visits, choking and gagging will be assessed by specific questions regarding frequency, a description of which food or drink and consistency was involved, how many times it occurred and maneuvers used to resolve the episode. Researchers will be trained to make sure they are able to distinguish a gagging event from a choking event while applying the questionnaire. We also hope the video examples shown to participants during the first intervention will help them distinguish between the two when reporting events to the researchers.

### Dietary assessment

At the 9-month and 12-month visits, trained researchers will administer a 24-h dietary recall (referring to the preceding day) designed to estimate the quantity, type, brands, time of the day, and cooking methods (including a description of any recipes used, with amounts of raw ingredients) of all foods and drinks consumed by the infant. If the mother considers the previous day to have been atypical, she can substitute another day of the week that was more similar to the family’s eating routine.

The consistency of the food or drink (puréed, mashed, diced or whole), who fed the child (parent, child, or both), iron supplementation (including type, brand, time of day offered, and the amount taken) will also be collected.

All diet records will be entered into the dietary analysis in nutrition software to analyze energy, protein, fat, carbohydrate, iron intake and other micronutrients.

### Biochemical assessment

At the 12-month visit, 6 mL of blood will be drawn from each child by venipuncture, preferably from the cubital vein. Each aliquot will be divided into 3 different tubes for analysis, containing 3 mL of blood each: namely: blacktop with no gel, for ferritin; and lavender top, containing the chelating agent ethylenediaminetetraacetic acid (EDTA), for complete blood count.

After blood collection, a pressure dressing will be placed at the puncture site and children will be instructed not to bear weight or apply force with the punctured arm for at least an hour [39].

If the child has an evident inflammatory or infectious condition at the time of collection, the procedure will be postponed to 14 days after remission of symptoms, in order to avoid possible momentary changes in laboratory parameters due to the disease.

#### Adverse events

We intend to monitor any adverse event caused by the trial participation. Presence of choking incidents will be identified in the questionnaires along with possible growth faltering, identified during the 9- and 12-month anthropometric measurement sessions. We will pay special attention to monitor multiple occurrences of adverse events in a specific participant as well as a high prevalence of any adverse event in multiple participants.

### Saliva collection and DNA extraction

Total DNA will be extracted from samples of oral mucosal cells present in saliva, collected with a sterile swab. The samples will be stored at − 20 °C in their collection tubes until processing. DNA extraction will be performed with a QIAamp DNA Mini Kit, which produces samples with high purity and yield. Quantification will be performed in an L-Quant system (Loccus).

### Analysis of polymorphisms

Nine SNPs previously associated with feeding preferences will be analyzed: rs1726866, rs713598, and rs10246939, located in the candidate gene *TAS2R38*; rs1376251, located on *TAS2R50*; rs846672, located at *TAS2R16*; rs35874116 and rs9701796, located on *TAS1R2*; and rs307355 and rs35744813, located in *TAS1R3*. Real-time PCR analysis will be used to determine the genotypes and polymorphisms through TaqMan® SNP genotyping assays, using the QuantStudio 3 Real-Time PCR System.

### Flavor acceptance test

The test will be performed as proposed by Schwartz, Issanchou, and Nicklaus (2009). Children will be offered solutions tasting of each of the predominant basic flavors: sweet, salty, sour, bitter, and umami [70]. The solutions will consist of mineral water and flavorings whose molecules can be easily found in the amniotic fluid during pregnancy, in breast milk, infant formula, or in complementary foods: lactose for sweet taste, sodium chloride for salty taste, urea for bitter taste, citric acid for sour taste, and monosodium glutamate for umami flavor.

Concentrations will be titrated to generate moderate flavor intensity by diluting 3.42 g of lactose, 0.25 g of sodium chloride, 0.057 g citric acid, or 0.085 g monosodium glutamate in 50 mL of mineral water (0.16 g urea will be diluted in 15 mL of water). Parents will be instructed to offer milk or water to the child 1 h before the test, and no more food or drink thereafter, to limit the variability of thirst and hunger and its potential impact on the acceptance of different flavors. The solutions will be offered in a container to which the child is accustomed (bottle or glass), as follows: one solution will be offered for 45 s, followed by a 15-s break; another 45-s offer of solution, another 15-s pause; an offer of water, followed by a 1- to 2-min pause before the next solution is offered. The test will be filmed for later analysis on a hedonic scale.

The solutions will be offered by a blinded researcher and weighed before and after the test. Flavor acceptance will be evaluated two ways: calculation of ingested volume and hedonic scale. The latter will be based on the child's facial and body reactions, on a scale of 5 points: (1) strong rejection, (3) mild rejection, (3) neutral reaction, (4) mild acceptance, and (5) strong acceptance.

### Oral health evaluation

At the 12-month visit, the visible biofilm and gingival bleeding will be recorded for each tooth surface [71]. With a clean dental surface after professional toothbrushing, the dental caries assessment will be performed using the International Caries Detection and Assessment System to detect Early Childrenhood Caries [72].

Primary dentition development and occlusal pattern will be determined by two previously calibrated examiners and the following variables evaluated: presence or absence of deep overbite, open bite, anterior crossbite, posterior cross bite, and overjet [73].

### Quality control

Each mother-child pair will receive a specific identification code number, corresponding to the questionnaires and examinations performed, to maintain the anonymity of the participants. To ensure the information provided to participants is standardized, the following procedures will be adopted:

Standard operating procedures: all the initial information given to explain the study will be saved in a document to be forwarded to the interested mothers. A flowchart of interventions with topics to be followed will be provided to ensure that investigators always address the same subjects.

Observed home visits: research staff will randomly be chosen to be accompanied by other investigators during some visits, to check for adherence to operating procedures.

Training: periodically, researchers will be trained to apply food recalls, anthropometric measurements, and questionnaires.

Data collection: 10% of the sample will be subject to a quality call to ascertain whether the interventions are being conducted as proposed. These preliminary results will be communicated to all researchers responsible for the interventions, in order to assess the needing to optimize the information-oriented interventions. If there is a need for improvement, this sample will receive reinforcement information regarding the complementary feeding method.

Data audits: Inter-evaluator TEM which consists of repeated anthropometric measurements, will be calculated annually on a sample of 10 participants by the research staff who are responsible for obtaining anthropometric measurements.

If any modification in the study protocol is necessary, a notice will be sent to the Ethics Committee of the HCPA and the Universidade Federal do Rio Grande do Sul (UFRGS), the ReBEC, as well as the researchers and the participant families.

The researchers will carry out preliminary analyzes throughout the study period, informing the responsible professors about its progress. The professors will make the final decision to complete the trial, after the inclusion of all participants, according to the sample calculation. When applicable, study updates will be added to the Brazilian Clinical Trials Registry (REBeC). Any situation unexpected by the survey will be evaluated individually. All professors and researchers will be notified and, if necessary, the family will be referred to their health professional or pediatrician.

The main results will be reported to the families at the end of the data collection, via email, and the trial results will be reported through presentations in congresses and seminars, and publications of articles.

### Statistical analysis

The database will be prepared in SPSS (Statistical Package for the Social Sciences) version 21.0, with double entry and validation. Descriptive analyses of continuous and categorical variables will be performed, in the isolated 10% quality assurance sample and in the total sample. Parametric data will be expressed as mean ± standard deviation. All analyses will be conducted by the intention-to-treat principle. The primary analysis will determine whether PLW, BLISS, or the mixed method results in differences in child growth *z*-scores at 12 months of age. Regression analyses will be used to analyze secondary outcomes, including iron intake and status. We will consider adjusting plasma ferritin where there is evidence of infection or inflammation, as indicated by elevated CRP. Statistical significance will be accepted at *p* < 0.05.

## Discussion

Adequate nutrition in early childhood has the potential to influence health consequences in later life. With growing recognition of the importance of nutrition in the early stages of life for health and discussion as to the best method for introducing complementary foods, investigation of best feeding practices at this stage should be a high priority. Although other authors have published studies of food introduction methods [18, 52], none has been conducted in the Brazilian population with this approach. Furthermore, to the best of our knowledge, this is the first study that will use a test kitchen to give mothers the opportunity of experiencing, in real-time, meals being prepared according to the standardized CF method to which they will be allocated. Additional pioneering contributions of this study will be reporting on the potential association of food introduction practices with prevalence of functional constipation, eating difficulties, oral health, and flavor outcomes.

Our findings will bridge the research gap on complementary feeding methods, with potential impact on short- and long-term health outcomes. Finally, we expect our findings will help further investigations and similar studies in this field.

## Trial status

Protocol version number: Brazilian Clinical Trials Registry (ReBEC): RBR- 229SCM number U1111-1226-9516. Date recruitment began: 14/03/2019. Approximate date when recruitment will be completed: 15/03/2022.

## Data Availability

The datasets used and/or analyzed during the current study are available from the corresponding author on reasonable request.

## Abbreviations

BEBQ: Baby Eating Behavior Questionnaire
BLISS: Baby-Led Introduction to SolidS
BLW: Baby-Led Weaning
CEBQ: Children Eating Behavior Questionnaire
CF: Complementary feeding
CFSQ: Caregiver Feeding Style Questionnaire
CRC: Clinical Research Center
CRP: C-reactive protein
DEBQ: Dutch Eating Behavior Questionnaire
EDTA: Ethylenediamine tetraacetic acid
FC: Functional constipation
HCPA: Hospital de Clínicas de Porto Alegre
IDA: Iron deficiency anemia
MCH-FS: Montreal Children Hospital Eating Scale
PLW: Parent-Led Weaning
ReBEC: Brazilian Clinical Trials Registry
SPSS: Statistical Package for the Social Sciences
UFRGS: Universidade Federal do Rio Grande do Sul
WHO: World Health Organization
