# Correction to: Complementary feeding methods in the first year of life: a study protocol for a randomized clinical trial

**DOI:** 10.1186/s13063-021-05824-2

**Published:** 2021-11-18

**Authors:** Leandro Meirelles Nunes, Jordana Führ, Christy Hannah Sanini Belin, Paula Ruffoni Moreira, Renata Oliveira Neves, Mariana Lopes de Brito, Lorenzo Andreazza Morando, Adriela Azevedo Souza Mariath, Erissandra Gomes, Juliana Rombaldi Bernardi

**Affiliations:** 1 Graduate Program in Child and Adolescent Health, Universidade Federal do Rio Grande do Sul (UFRGS) Medical School, Porto Alegre, Brazil; 2 Neonatology Section, Hospital de Clínicas de Porto Alegre (HCPA), Porto Alegre, Brazil; 3 Graduate Program in Food, Nutrition and Health, Universidade Federal do Rio Grande do Sul (UFRGS) Nutrition Department, Medical School, Porto Alegre, Brazil; 4 Department of Surgery and Orthopedics, Universidade Federal do Rio Grande do Sul (UFRGS) Dentistry School, Porto Alegre, Brazil; 5 Nutrition Department, Universidade Federal do Rio Grande do Sul (UFRGS), Hospital de Clínicas de Porto Alegre (HCPA), Porto Alegre, Brazil

**Correction to: Nunes (2021).**

**https://doi.org/10.1186/s13063-021-05647-1**

Following the publication of the original article [1], we were notified that the wrong birth weight was introduced during Production: 3.500 g (incorrect) instead of 2.500 g (correct).

The original article has been corrected.
